# 

**Published:** 1882-01

**Authors:** 


CONTENTS.

| | | | |
|---|---|---|---|
| Foods, and How to Use Them | 1 | Consumption Cure | 25 |
| Air | 3 | Practical Knowledge | 26 |
| Hygiene | 4 | Eight to Sixteen | 28 |
| Accidents | 5 | Farmers and Citizens | 29 |
| About Dinner Hour | 7 | Over-eating | 31 |
| Deterioration of the Eye | 8 | "Going It" | 33 |
| "'Tis Easy to Die" | 9 | A Dangerous Curiosity | 34 |
| The Sago Trees | 10 | The Feet in Winter | 35 |
| An Iowa Girl's Ambition | 11 | Baths and Bathing | 36 |
| Pleasantly Flavored | 12 | Marvels of Man | 37 |
| Paris Crime | 13 | Recreation | 39 |
| Midnight in a Prison | 14 | Health | 40 |
| The Teeth | 15 | Drinking Water | 41 |
| The Nature of Consumption | 21 | For the Farm and Family | 44 |
| Hereditary Disease | 21 | My Life (Poetry) | 47 |
| Small Pox | 23 | Poison Sumac | 49 |

